# The transcription factor EBF1 non-cell-autonomously regulates cardiac growth and differentiation

**DOI:** 10.1242/dev.202054

**Published:** 2023-10-24

**Authors:** Eugene E. Kim, Akshay Shekhar, Jayalakshmi Ramachandran, Alireza Khodadadi-Jamayran, Fang-Yu Liu, Jie Zhang, Glenn I. Fishman

**Affiliations:** Leon H. Charney Division of Cardiology, NYU Grossman School of Medicine, New York, NY 10016, USA

**Keywords:** EBF1, Purkinje cell, Heart, Hyperplasia, Transcription factor, Mouse

## Abstract

Reciprocal interactions between non-myocytes and cardiomyocytes regulate cardiac growth and differentiation. Here, we report that the transcription factor *Ebf1* is highly expressed in non-myocytes and potently regulates heart development. *Ebf1*-deficient hearts display myocardial hypercellularity and reduced cardiomyocyte size, ventricular conduction system hypoplasia, and conduction system disease. Growth abnormalities in *Ebf1* knockout hearts are observed as early as embryonic day 13.5. Transcriptional profiling of *Ebf1-*deficient embryonic cardiac non-myocytes demonstrates dysregulation of Polycomb repressive complex 2 targets, and ATAC-Seq reveals altered chromatin accessibility near many of these same genes. Gene set enrichment analysis of differentially expressed genes in cardiomyocytes isolated from E13.5 hearts of wild-type and mutant mice reveals significant enrichment of MYC targets and, consistent with this finding, we observe increased abundance of MYC in mutant hearts. EBF1-deficient non-myocytes, but not wild-type non-myocytes, are sufficient to induce excessive accumulation of MYC in co-cultured wild-type cardiomyocytes. Finally, we demonstrate that BMP signaling induces *Ebf1* expression in embryonic heart cultures and controls a gene program enriched in EBF1 targets. These data reveal a previously unreported non-cell-autonomous pathway controlling cardiac growth and differentiation.

## Teaser

**Highlighted Article:** The transcription factor EBF1 is expressed in non-myocytes in the heart, where it regulates the proliferation and differentiation of cardiomyocytes in a non-cell-autonomous manner.

## INTRODUCTION

Intercellular interactions play essential roles in regulating the growth and differentiation of the three primitive heart layers: the endocardium, myocardium and epicardium (Brutsaert, 2003; Hsieh et al., 2006; Saint-Jean et al., 2019). Early in heart development, during the tube stage before looping, the endocardial cells are the predominant non-myocyte cell (NMC) population, and their appearance precedes that of the epicardium. Subsequently, the endocardium plays a crucial role in the development of the underlying trabecular myocardium (Del Monte-Nieto et al., 2018; Lee et al., 1995; Pentassuglia and Sawyer, 2009), at least in part through the elaboration of signaling molecules such as neuregulin 1 (NRG1). Reciprocally, trabecular myocyte-derived factors, such as bone morphogenetic protein 10 (BMP10), induce an endocardial cell fate from *Nkx-2.5*^+^ cardiac precursors and are indispensable for proper trabecular development and cardiac growth (Barron et al., 2000; Mikryukov et al., 2021). Later in development, after ventricular septation, epicardium- and endocardium-derived cells migrate transmurally to populate the cardiac interstitial space with a variety of NMC types that play key roles in cardiomyocyte maturation (Carmona et al., 2020; Eralp et al., 2006; Goodyer et al., 2019; Hortells et al., 2020; Quijada et al., 2020; Wang et al., 2020a; Wu et al., 2012; Zhang et al., 2018). Additionally, neural crest cells also contribute to the NMC cell population of the heart (Erhardt et al., 2021). Crucial to the interdependence of NMCs and cardiomyocytes are gene regulatory networks in which key transcription factors govern the signal-dependent gene activation and repression necessary for proper cellular development.

Sequence-based computational analyses have identified a substantial number of incompletely characterized cis-regulatory elements (CREs) and their predicted cognate transcription factors (TFs) in the human heart (Lee et al., 2018). Among these is the basic-helix-loop-helix (bHLH) transcription factor early B-cell factor 1 (*Ebf1*) (Lee et al., 2018). Since its initial description as a key factor essential for B-cell differentiation (Hagman et al., 1993; Lin and Grosschedl, 1995), *Ebf1* has been implicated in the development of kidney glomeruli (Fretz et al., 2014), neurons (Garel et al., 2002), bone (Zee et al., 2013) and adipose tissue (Jimenez et al., 2007). Despite enrichment of EBF1 CREs in cardiac tissue, to date no role for this TF in heart formation, function or disease pathogenesis has been reported. EBF1 is particularly interesting, as it is a primordial transcription factor with orthologs traced back throughout metazoan evolution (Dubois and Vincent, 2001). Initially discovered as an essential transcription factor for B lymphocyte development (Boller and Grosschedl, 2014), *Ebf1* acts to both establish and maintain B-cell specification (Nechanitzky et al., 2013). Described as a ‘pioneer factor’, *Ebf1* influences cell fate determination through its ability to induce lineage-specific changes in chromatin accessibility (Boller et al., 2016), allowing gene poising, activation or repression (Treiber et al., 2010). In the current work, we describe a role for *Ebf1* in the developing heart, including formation of the specialized ventricular conduction system.

## RESULTS

### *Ebf1*-deficient hearts exhibit abnormal cardiac structure

**Fig. 1.**
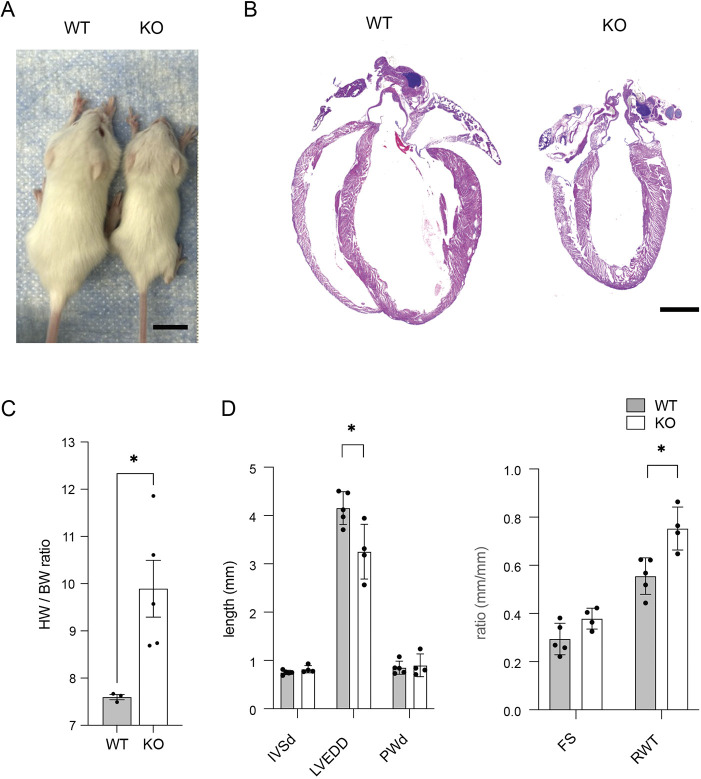
**Abnormal cardiac form in** ***Ebf1*** **knockout mice.** (A) *Ebf1* wild-type and knockout mice at postnatal day 21. (B) Hematoxylin and Eosin-stained images of postnatal day 21 wild-type and KO heart sections. (C) Heart weight to body weight ratio measurements in wild-type and KO mice. (D) Echocardiographic measurements of the left ventricular wall thickness (IVSd, interventricular septum diastole; PWd, posterior wall diastole), left ventricular end diastolic diameter (LVEDD), fractional shortening (FS) and relative wall thickness (RWT) in *Ebf1* wild-type and KO mice. Scale bars: 1 cm in A; 1 mm in B. *P<0.05 (unpaired *t*-test). WT, wild type.

To investigate the phenotypic consequences of *Ebf1* loss of function on cardiac structure, we performed histological examination of hearts isolated from postnatal day 21 wild-type and *Ebf1*-deficient (KO) mice. We observed that wild-type and KO mice were indistinguishable at birth, but by the time of weaning, KOs were overtly runted (Fig. 1A), as previously reported (Lin and Grosschedl, 1995). Although the hearts of KO mice were also significantly smaller than wild-type littermates (Fig. 1B), heart weight to body weight ratios were significantly increased (Fig. 1C). Histological examination of Hematoxylin and Eosin-stained sections revealed reduced chamber size, but similar ventricular wall thickness between KO and wild-type hearts (Fig. 1B), suggesting relative hypertrophy or hyperplasia in the mutant hearts. This finding was confirmed with echocardiography, which demonstrated smaller left ventricular chamber size, as assessed by end diastolic diameter, but no significant difference in wall thickness, resulting in an increase in relative wall thickness (Fig. 1D). From these data, we conclude that EBF1 deficiency results in overall runting, with an increase in heart size relative to body size and an increase in relative left ventricular wall thickness.

### Myocardial hyperplasia in *Ebf1*-deficient hearts

**Fig. 2.**
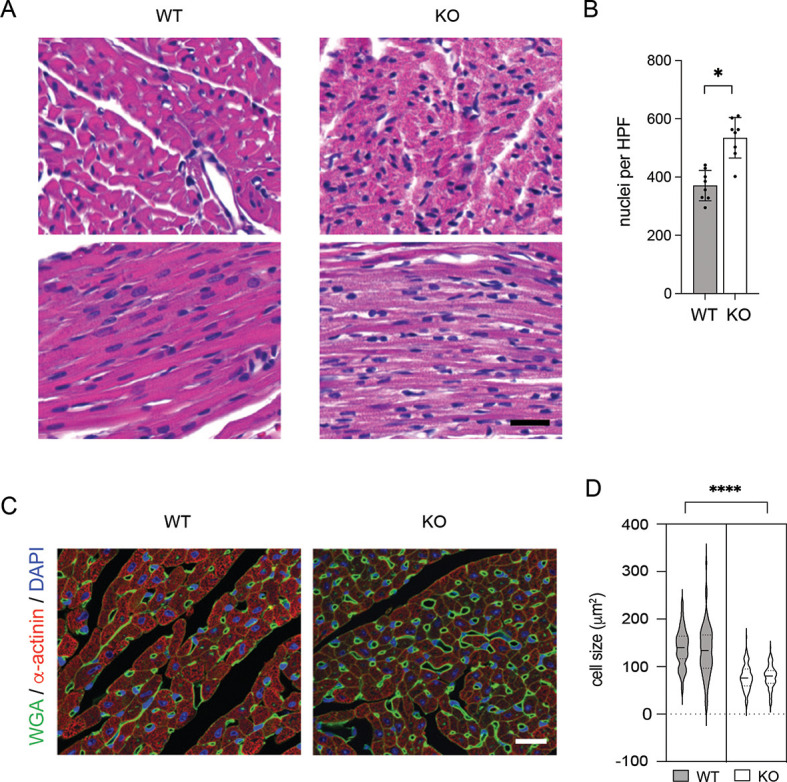
**Myocardial hyperplasia in** ***Ebf1*** **knockout mice.** (A) High-magnification Hematoxylin and Eosin-stained images of the left ventricular free wall with cardiomyocytes in short axis (upper panels) and long axis (lower panels). (B) Quantitative image analysis of nuclei per high power field in *Ebf1* wild-type and KO cardiomyocytes. (C) Immunofluorescence staining of wheat germ agglutinin (WGA), α-actinin and DAPI. (D) Violin plot of cell size distribution in *Ebf1* wild-type and KO hearts. Each violin represents a separate heart. Scale bars: 20 mm. *P<0.05, ****P<0.001 (unpaired *t*-test). WT, wild type.

To investigate the basis for increased relative wall thickness in *Ebf1* KO hearts, high-magnification histological analysis was performed that revealed a marked increase in nuclei per area in the mutant hearts (Fig. 2A,B). Additionally, cell size characterization using the membrane-delineating marker wheat germ agglutinin demonstrated significantly smaller cell size in knockout hearts (Fig. 2C,D), indicative of hyperplasia as the basis for the increase in relative wall thickness. From these data, we conclude that EBF1 deficiency results in myocyte hyperplasia and smaller cell size.

### Persistent postnatal cardiomyocyte proliferation in Ebf1 knockout hearts

**Fig. 3.**
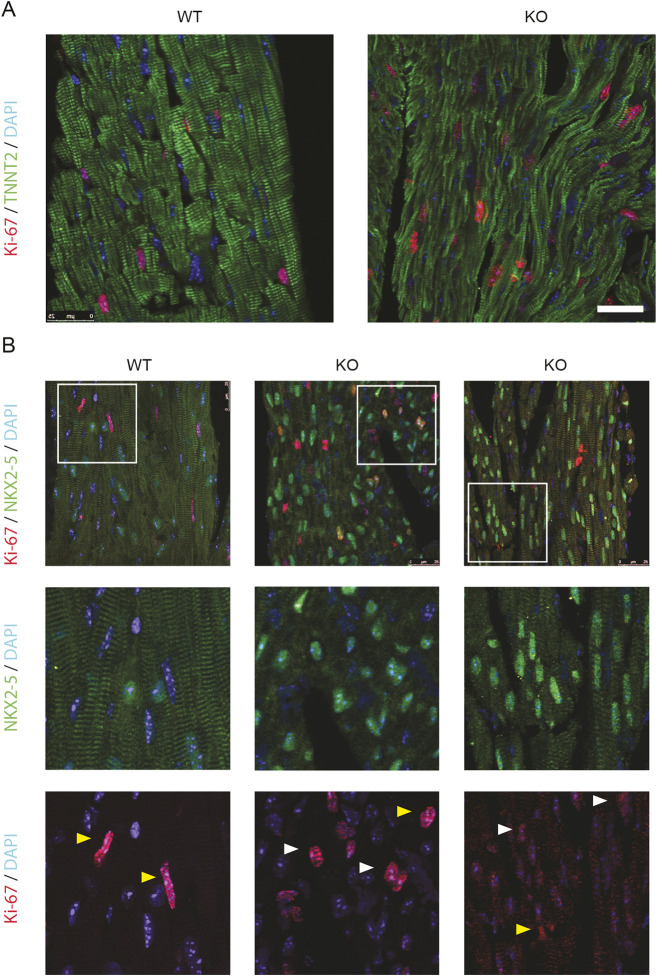
**Persistent postnatal cardiomyocyte proliferation in *Ebf1* knockout mice.** (A) Immunostaining of heart sections from P21 wild-type and knockout mice for KI-67, TNNT2 and DAPI. (B) Immunostaining of heart sections from P21 wild-type and two separate knockout mice for KI-67, NKX2.5 and DAPI. Lower panels show high-magnification images of the areas outlined in the top panels. White arrowheads indicate nuclei positive for both Ki-67 and NKX2.5. Yellow arrowheads indicate nuclei positive for Ki-67 only. Scale bar: 25 µm. WT, wild type.

The smaller cardiomyocyte size and paler eosin staining seen on Hematoxylin and Eosin (Fig. 2A) in *Ebf1* KO mice suggested cellular immaturity. To investigate this finding further, we performed immunostaining for the sarcomeric protein troponin T2 (TNNT2), and found that sarcomeric structure was indeed very disorganized and appeared immature (Ahmed et al., 2022) in KO animals (Fig. 3A). During the postnatal period, cardiomyocytes exit the cell cycle and undergo terminal differentiation and maturation. Given the apparent abnormalities in cell morphology that suggest immaturity, we sought to assess whether *Ebf1* KO cardiomyocytes appropriately exited the cell cycle. Staining was therefore performed for the proliferation marker Ki-67, along with the cardiac sarcomeric protein troponin T2 (TNNT2) (Fig. 3A) and the cardiac transcription factor NKX2.5 (Fig. 3B) at postnatal day 21, well into the postmitotic phase for cardiomyocytes. This immunostaining demonstrated that proliferation in the wild-type animals was restricted to NMCs. In contrast, nuclear Ki-67 could be detected to varying degrees in KO cardiomyocytes (Fig. 3B). These data suggest a role for *Ebf1* in regulating cardiomyocyte maturation and appropriate postnatal cell cycle exit.

### Conduction system defects in *Ebf1* mutant mice

**Fig. 4.**
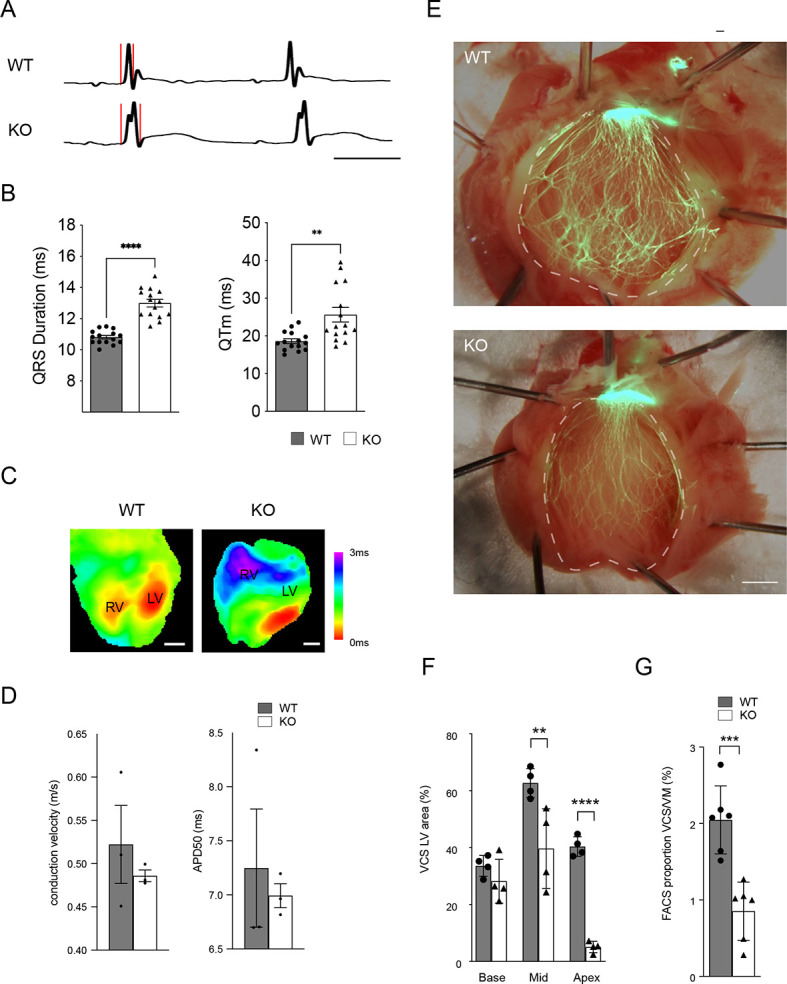
**Abnormal ventricular conduction system form and function in *Ebf1*-null mice.** (A) ECG tracings from wild-type and *Ebf1* KO animals shows QRS duration prolongation. Red lines indicate the beginning and end of the QRS complex. Scale bar: 50 ms. (B) ECG intervals in wild-type and *Ebf1* KO animals. QRS and QT intervals are significantly prolonged. (C) Optical maps of the anterior epicardial surface show abnormal patterns of ventricular depolarization in KO animals. (D) Bar graph of conduction velocity and action potential duration at 50% repolarization (APD50) measured from activation maps of wild-type and *Ebf1* KO animals. (E) Whole-mount images of left ventricular endocardial surface of wild-type and *Ebf1* KO mice using the *Cntn2-EGFP* reporter. (F,G) Quantitative image analysis of VCS area as a percentage of left ventricular endocardial surface (F) and FACS analysis of the proportion of GFP^+^ myocytes (G) from postnatal day 21 *Ebf1* wild-type and KO hearts in *Cntn2-EGFP* background. Scale bars: 1 mm. **P<0.01, ***P<0.001, ****P<0.0001 (unpaired *t*-test). WT, wild type.

Ventricular conduction system (VCS) cells comprise a group of highly specialized cardiomyocytes derived from the trabecular myocardium, a zone that normally proliferates more slowly than compact zone myocardial cells (Park et al., 2013). To determine whether the generalized proliferative defect observed in *Ebf1*-deficient hearts might be especially impactful in the VCS, we performed surface electrocardiography on adolescent (P21-P42) wild-type and EBF1 KO mice. This analysis revealed significant prolongation of the QRS interval in knockout (KO) mice compared with wild-type controls, which is indicative of impaired conduction through the specialized VCS and/or slow, dyssynchronous ventricular depolarization (Fig. 4A,B). We also noted a prolonged QT interval, indicating delayed repolarization, an expected consequence of aberrant ventricular depolarization. To gain a more detailed assessment of ventricular activation patterns, we performed optical mapping of isolated hearts using voltage-sensitive dyes. In wild-type animals, the expected biventricular apical activation pattern was seen in all animals studied (*n*=3), indicative of normal conduction within the VCS and near simultaneous activation of the left and right ventricles (Fig. 4C). In contrast, in all KO animals (*n*=3), there was failure of right-sided breakthrough and delayed depolarization of the right ventricle, suggestive of conduction block within the right bundle branch (Fig. 4C). Conduction velocity and action potential duration were not significantly different in the KO animals (Fig. 4D).

To directly examine the basis for the conduction defects observed in EBF1 mutant mice, we bred them with the *Cntn2-EGFP* reporter line (Maass et al., 2015), which visualizes expression of the ventricular conduction system, and performed whole-mount imaging of EGFP. This analysis revealed profound hypoplasia of the VCS in mutant hearts (Fig. 4E). The paucity of Purkinje cells within the VCS was particularly pronounced toward the apex of mutant hearts (Fig. 4F). Consistent with the hypoplastic VCS phenotype, FACS analysis of isolated cardiomyocytes from wild-type and mutant hearts demonstrated a significant reduction in the percentage of Purkinje cells compared with ventricular myocytes in the KO background (Fig. 4G). Taken together, these findings suggest that loss of function of *Ebf1* results in aberrant cardiomyocyte differentiation, including highly specialized Purkinje cells of the VCS.

### Developmental defects in *Ebf1*-deficient embryonic hearts

**Fig. 5.**
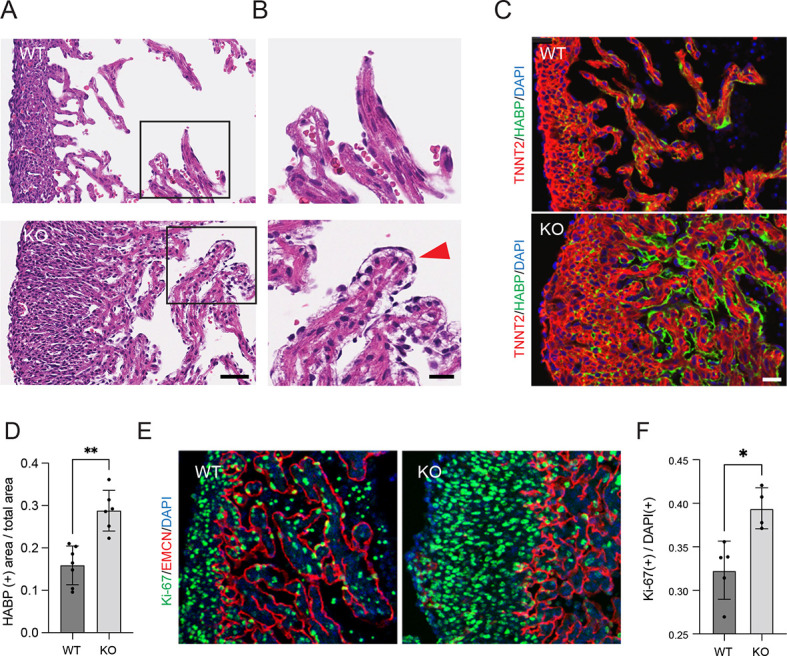
**EBF1 regulates myocardial architecture and proliferation during heart development.** (A) Hematoxylin and Eosin staining of transmural sections from the left ventricles of E13.5 wild-type and *Ebf1* mutant hearts at the midventricular level. (B) Expanded views of areas outlined in A. Red arrowhead indicates extracellular matrix bubble. (C) Immunostaining of the extracellular matrix using hyaluronic acid-binding protein (HABP) and of cardiomyocytes using troponin T2 (TNNT2) in wild-type and *Ebf1* mutant hearts. (D) Quantification of the ratio of HABP^+^ area to total myocardial area in wild-type and *Ebf1* mutant hearts. (E) Immunostaining for the Ki-67 proliferation marker along with the endocardial marker endomucin (EMCN) in wild-type and *Ebf1* mutant hearts. (F) Quantification of the ratio of Ki-67^+^ nuclei to total nuclei in wild-type and *Ebf1* mutant hearts. Scale bars: 25 µm in A,C,E; 10 μm in B. *P<0.05, **P<0.01 (unpaired *t*-test). WT, wild type.

Given the marked abnormalities in cardiac structure and function in adult *Ebf1*-deficient hearts, we examined E13.5 embryonic hearts to investigate whether there might be a developmental basis for the observed phenotype. Even at this early stage, we observed marked hypercellularity in *Ebf1*^−/−^ ventricles, with expansion of both the compact and trabecular myocardium (Fig. 5A). Additionally, the trabeculae carnae in the KO hearts displayed abnormal architecture, with crowding of the ventricular lumen, excessive extracellular matrix deposition and the presence of persistent endocardial bubbles around the tips of trabeculae (Fig. 5B). To confirm that the expanded intercellular spaces seen between the myocytes and endocardial cells in the KO hearts was related to ECM deposition, staining with hyaluronic acid-binding protein (HABP) was performed. This analysis demonstrated a marked excess of hyaluronic acid deposition in mutant hearts (Fig. 5C,D). Although this pattern is seen in the early stages of normal trabecularization (Del Monte-Nieto et al., 2018), by embryonic day 13.5, wild-type hearts typically demonstrate loss of the ECM bubble and close apposition of the endocardium to the trabecular myocytes. In addition to this trabecular defect, we also observed a difference in cellular proliferation, as visualized by staining for Ki-67 along with the endocardial marker endomucin (EMCN) (Fig. 5E). *Ebf1*^−/−^ hearts showed a significant increase in Ki-67-positive nuclei compared with control hearts (39.4±0.02% versus 32.3±0.03%; *P*=0.0159) (Fig. 5F). From these studies, we conclude that loss of function of *Ebf1* results in dysregulated cardiomyocyte proliferation, as well as defects in trabecular maturation. This latter finding is consistent with the paucity of highly differentiated Purkinje cells observed in the VCS of adult mutant hearts.

### Cardiac expression of EBF1 is restricted to NMCs

**Fig. 6.**
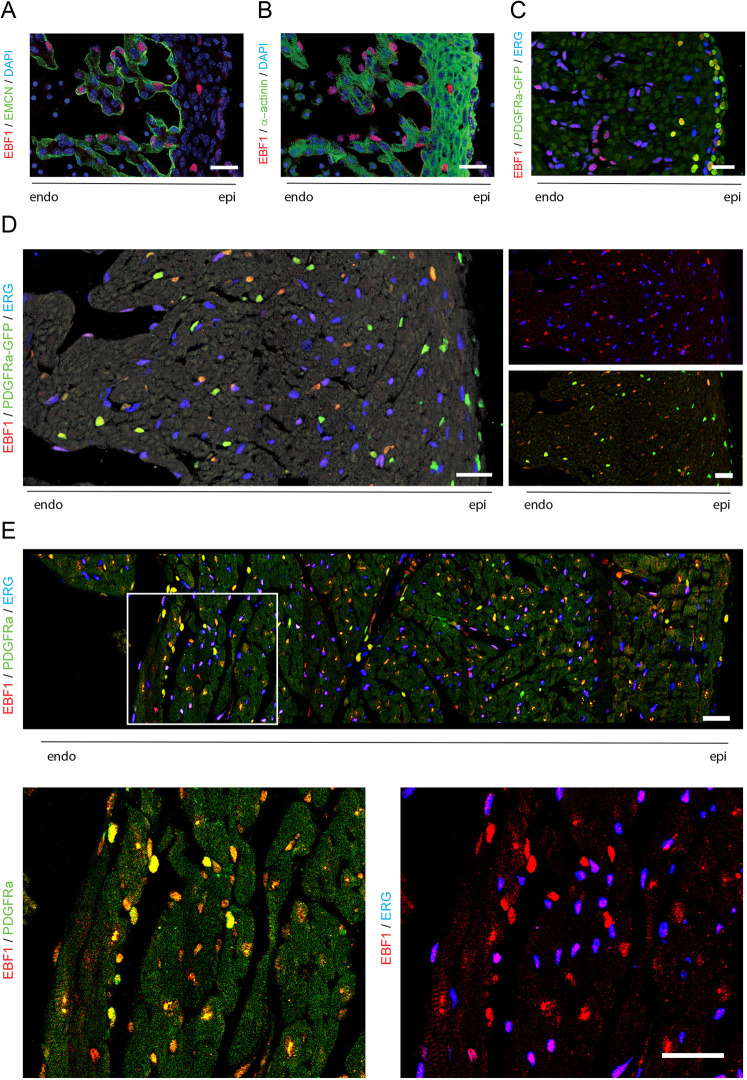
**EBF1 expression in the murine heart.** (A,B) Immunofluorescence of EBF1 co-stained with EMCN (A) or the myocyte marker α-actinin (B) in E13.5 embryonic heart sections. (C-E) Immunofluorescence of EBF1 co-stained with the endothelial marker ERG and the fibroblast/smooth muscle cell marker PDGFRα in heart sections at (C) E13.5, (D) postnatal day 1 and (E) postnatal day 21. High-magnification images of areas outlined in E are shown underneath. All images are at the mid level of the left ventricle. Scale bars: 25 µm.

Expression of EBF1 in the developing and postnatal murine heart was examined using immunolocalization. At E13.5, during the initial stages of coronary vasculature development and ingrowth of epicardium-derived interstitial cells, robust EBF1 expression was seen in endocardial cells, which were identified by expression of endomucin (EMCN) (Fig. 6A). Unexpectedly, no expression was observed in cardiomyocytes identified by expression of α-actinin (Fig. 6B). We also observed EBF1 expression within scant interstitial cells in the compact myocardium as well as in cells near the epicardium. These cells were characterized by the expression of PDGFRα, a marker of epicardium-derived cells, including cardiac fibroblasts and pericytes (Quijada et al., 2020) (Fig. 6C). Closer inspection of the EBF1-expressing cells near the epicardium reveals their location in the immediate subepicardial layer rather than the true epicardium and identifies them as epicardium-derived progenitor cells (EPDCs) (Quijada et al., 2020). At P1, when the myocardium is further populated with coronary vascular and interstitial cells, EBF1 expression was seen in nearly all NMC types (Fig. 6D). In addition to endocardial and EPDC expression, EBF1 was detected in PDGFRα-expressing interstitial and perivascular cells, and in a subset of ERG-expressing endothelial cells (Fig. 6D). At this stage, the highest expression of EBF1 was seen in the PDGFRα-expressing cells, with weaker endocardial and vascular endothelial expression. A similar expression pattern was also observed at P21 (Fig. 6E), with EBF1 expression restricted to cells expressing one of these two non-myocyte markers. From these data, we conclude that EBF1 expression in the heart is restricted to NMCs. The absence of EBF1 expression in cardiomyocytes suggests that the observed defects in cardiomyocyte proliferation and cell size likely arise through a non-cell-autonomous mechanism.

### *Ebf1* regulates Polycomb-group protein target gene transcription

**Fig. 7.**
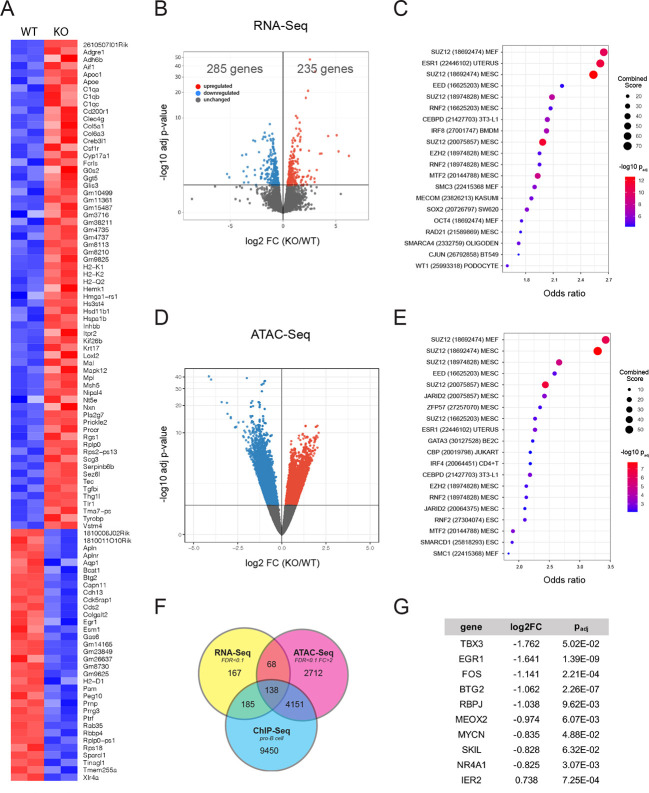
**Gene profiling and chromatin analysis of embryonic *Ebf1^−/−^* non-myocytes.** (A) Heatmap of the top 100 DEGs between E13.5 non-myocyte cells (NMCs) isolated from wild-type and KO hearts. (B) Volcano plot of DEGs. (C) Top 20 ChIP-Seq gene sets identified using ChEA3 analysis of DEGs. (D) Volcano plot of differentially accessible regions (DARs) identified by ATAC-Seq in wild-type and KO NMCs. (E) Top 20 ChIP-Seq gene sets identified using ChEA3 analysis of genes near DARs. (F) Venn diagram showing the genes shared between RNA-Seq identified DEGs and ATAC-Seq identified DARs in KO NMCs, and previously validated *Ebf1* target genes. (G) Transcription factors shared between RNA-Seq, ATAC-Seq and ChIP-Seq analyses, along with relative expression in knockout cells and statistical significance.

To investigate the mechanisms responsible for the hyperproliferative phenotype seen in *Ebf1* knockout hearts, we first performed transcriptional profiling of KO cells from dissociated E13.5 hearts. At this stage of development, EBF1 expression is restricted largely to endocardial cells. After cell isolation using indirect immunolabeling for PECAM1 and FACS, gene profiling revealed 520 differentially expressed genes (DEGs): 285 transcripts were upregulated and 235 transcripts were downregulated at a FDR of 0.1 in the PECAM1^+^ EBF1 KO cells (Fig. 7A,B). Transcription factor-binding site analysis was then performed using ChEA3 (Keenan et al., 2019) and revealed significantly dysregulated expression of gene targets of the Polycomb-group (PcG) of proteins, Polycomb repressive complex 1 and 2 (PRC1 and PRC2), SUZ12, EED, EZH2, MTF2 and RNF2 (Fig. 7C). Given these findings, and the known role of EBF1 as a pioneer factor and chromatin modifier (Wang et al., 2020b), the chromatin landscape of non-myocytes isolated from wild-type and KO hearts at E13.5 was analyzed using ATAC-Seq. This analysis revealed 3940 genomic regions that were less accessible and 3156 genomic regions that were more accessible for a total of 7069 differentially accessible regions (DARs) in KO cells (FDR<0.1, FC>2) (Fig. 7D), consistent with the known role of EBF1 in enhancing chromatin accessibility. When mapped to the nearest gene, these DARs correspond to 3250 genes. Of these, 206 were also among the 558 DEGs identified in the transcriptional analysis (Fig. 7F). Transcription factor-binding analysis of the genes identified by ATAC-Seq revealed striking similarities to the analysis performed on the DEGs of knockout cells in that PRC1 and PRC2 target genes were significantly enriched (Fig. 7E). In particular, SUZ12 was noteworthy in the degree to which its target genes were enriched in both the RNA-Seq and ATAC-Seq analyses. When compared with a previously published ChIP-Seq dataset (Ungerbäck et al., 2015; GEO accession number GSM1695664), 138 of DEGs were shared between the RNA-Seq, ATAC-Seq and ChIP-Seq analyses (Fig. 7F), of which 10 are known transcription factors: BTG2, EGR1, FOS, IER2, MEOX2, MYCN, NR4A1, RBPJ, SKIL and TBX3. These genes were all significantly downregulated in EBF1-deficient NMCs (Fig. 6G). Diminished expression of *Tbx3* (−1.73±0.21 log2FC) was particularly notable given the established role of this transcriptional repressor in VCS development (Bakker et al., 2008; Mohan et al., 2018). Additionally, *Egr1* (−1.641±0.23 log2FC) has previously been implicated in differentiation and maturation in a variety of cellular contexts (Bléher et al., 2020; Dinkel et al., 1998; Veyrac et al., 2013), and has been used as a marker of cardiac fibroblast maturation (Wang et al., 2020a). Together these, data support a model in which EBF1 regulates the chromatin accessibility and expression of a gene network that includes transcription factors important for cellular development.

### MYC overexpression in *Ebf1*-deficient cardiomyocytes

**Fig. 8.**
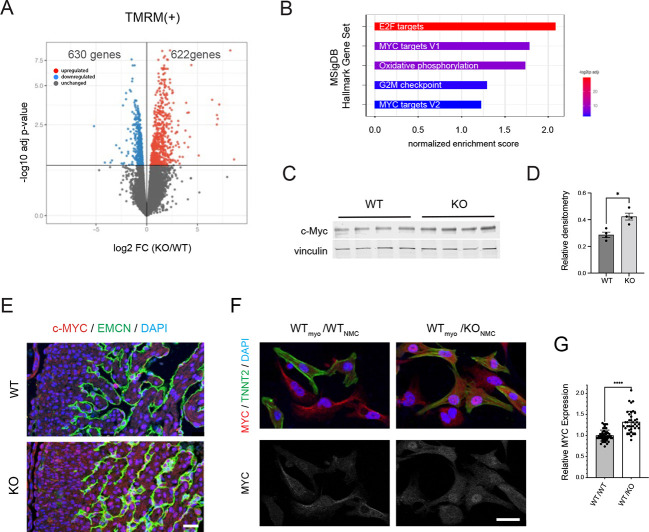
**MYC overexpression in embryonic *Ebf1^−/−^* myocytes.** (A) Volcano plot of DEG in KO cardiomyocytes (FDR=0.25). (B) GSEA analysis plots of top four gene sets enriched in KO cardiomyocytes. NES, normalized enrichment score. (C) Western blot of Myc in E13.5 ventricles from wild-type and *Ebf1* KO hearts. (D) Densitometry quantification of the western blot in C. (E) Immunofluorescence images of E13.5 wild-type and KO heart sections stained for MYC, EMCN and DAPI. (F) Immunofluorescence images of neonatal heart cell co-cultures of wild-type myocytes cultured with wild-type (WT_myo_/WT_NMC_) or KO (WT_myo_/KO_NMC_) NMCs stained for MYC, troponin T2 (TNNT2) and DAPI. (G) Quantification of nuclear MYC expression in neonatal myocytes cultured with wild-type or KO NMCs. *P<0.05, ****P<0.0001 (unpaired *t*-test). Scale bars: 25 µm. WT, wild type.

We next performed transcriptional profiling of wild-type and *Ebf1*-deficient cardiomyocytes isolated from E13.5 hearts using the mitochondrial dye tetramethylrhodamine, methyl ester (TMRM) and FACS. We found that expression of 622 transcripts was enriched and of 630 transcripts was diminished in the KO cardiomyocytes at an FDR of 0.1 when compared with wild-type cardiomyocytes (Fig. 8A). Gene set enrichment analysis (GSEA) of DEGs between wild-type and KO cardiomyocytes was performed and revealed significant enrichment of several gene sets in KO cardiomyocytes, including ‘E2F targets’ (NES: 2.11; FDR: 0.000), ‘MYC targets’ (NES: 1.76; FDR: 0.006) and ‘oxidative phosphorylation’ (NES: 1.73; FDR: 0.005) (Fig. 8B). MYC is a potent proto-oncogene that has been shown to regulate the expression of the E2F family of transcription factors (Leone et al., 1997) and cellular metabolism (Stine et al., 2015), suggesting a unifying mechanism to account for the increase in cell proliferation seen with *Ebf1* deficiency. To determine whether MYC was indeed dysregulated in *Ebf1* KO hearts, we performed western blots, which revealed increased expression in developing ventricles, consistent with the GSEA analysis (Fig. 8C,D). The expression of MYC was also assessed using immunofluorescence and revealed significantly higher expression in KO hearts (Fig. 8E). Interestingly, *Myc* transcripts were not significantly enriched in EBF1-deficient cardiomyocytes (217.2±47.4 versus 208.7±26.7 normalized counts; *P*=0.83), indicative of a post-transcriptional mechanism. These data suggest that an EBF1-dependent paracrine signaling pathway between non-myocytes and cardiomyocytes leads to aberrant MYC accumulation in the latter cell type. To test this hypothesis, we incubated wild-type cardiomyocytes with either wild-type NMCs or EBF1-deficient NMCs, and performed double immunofluorescent staining with antibodies directed against troponin T and MYC. We observed significantly increased nuclear MYC expression in the cardiomyocytes co-cultured with EBF1-deficient NMCs (Fig. 8F,G). At low to medium density, NMCs had little direct contact with neighboring cardiomyocytes, favoring a paracrine, rather than juxtacrine, mechanism. Taken together, these data indicate that loss of function of EBF1 in non-myocytes impacts cardiomyocyte proliferation and differentiation through non-cell-autonomous mechanism. Moreover, they implicate aberrant control of cardiomyocyte MYC abundance as a key determinant of the proliferative and developmental defects we observe, including hypoplasia of the ventricular conduction system.

### *Ebf1* expression is induced by BMPs

**Fig. 9.**
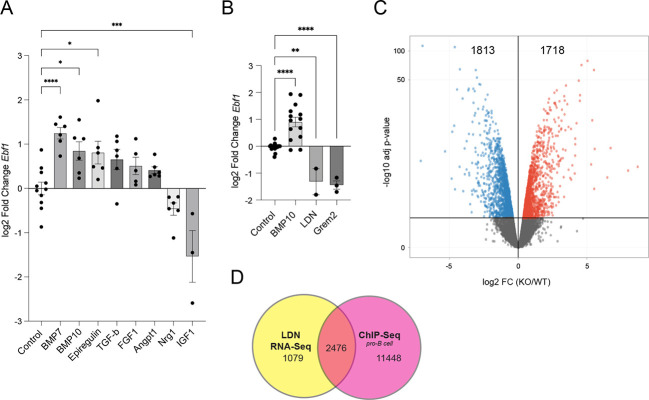
**BMP-mediated regulation of *Ebf1* expression in embryonic hearts.** (A) Bar graph of *Ebf1* expression in E9.5 heart cultures exposed to various ligands for 48 h. (B) Bar graph of *Ebf1* expression in E9.5 heart cultures exposed to control vehicle, BMP10, LDN or gremlin 2 for 48 h. (C) Volcano plot of DEGs in embryonic heart cultures exposed to LDN for 48 h. (D) Venn diagram of DEGs of LDN-treated hearts and EBF1 gene targets using published ChIP-Seq data (Ungerbäck et al., 2015) from pro B-cells. *P<0.05, **P<0.01, ***P<0.001, ****P<0.0001 (one way ANOVA).

The data above suggested that expression of EBF1 in NMCs in the developing heart is required to prevent aberrant cardiomyocyte proliferation. Therefore, to identify potential inducers of Ebf1 expression, we used an embryonic heart culture model system. We incubated E9.5 embryonic hearts with various ligands from the EGF, FGF, BMP, TGFβ and IGF growth factor families and measured *Ebf1* transcript abundance 48 h later by qPCR. Treatment with both BMP7 and BMP10 resulted in a significant increase in *Ebf1* expression (Fig. 9A). To assess whether BMP signal antagonism would affect *Ebf1* expression, we used the chemical inhibitor LDN193189 (LDN) or recombinant gremlin 2 (Grem2), both potent BMP signal antagonists. Separately applied, both treatments significantly repressed *Ebf1* accumulation to below control levels (Fig. 9B). LDN-treated hearts were also subjected to gene profiling. As anticipated, *Ebf1* was significantly repressed (−0.68±0.27 log2FC; *P*_adj_–0.05). A total of 3555 differentially expressed genes were identified, 1838 were downregulated and 1718 were upregulated at an FDR of 0.1 (Fig. 9C). Of these, 2476 (69%) are direct targets of EBF1 based on published ChIP-Seq data from pro B-cells (Ungerbäck et al., 2015) (Fig. 9D). Based on the presence of EBF1-binding sites in 13,924 genes, or in 28.58% of the known mouse genome, the enrichment of EBF1 gene targets among the DEGs in LDN-treated samples was highly significant (χ^2^–2939.58, *P*<0.0001), suggesting an important role for EBF1 in BMP signal transduction. Taken together, these data indicate that myocardial BMP expression in the developing heart may induce *Ebf1* in non-myocytes, which in turn signal back to the cardiomyocytes and modulate their proliferative activity.

## DISCUSSION

In the current study, we describe the role of the helix-loop-helix transcription factor *Ebf1* in cardiac development. Since its initial description in B-cells (Boller and Grosschedl, 2014), *Ebf1* has been implicated in the development of a variety of cell types and organ systems, including kidney (Fretz et al., 2014), nervous system (Dubois et al., 1998; Garcia-Dominguez et al., 2003; Garel et al., 1999), adipose tissue (Aåkerblad et al., 2002; Jimenez et al., 2007) and bone (Zee et al., 2013). In each of these contexts, failure of terminal cellular differentiation is a common feature. Given the recent observation that human cardiac tissue is enriched in cis-regulatory elements predicted to bind EBF1 (Lee et al., 2018), in this study we sought to determine the role of this bHLH transcription factor in cardiac development and physiology. We observed multiple defects in EBF1 KO hearts, including marked myocardial hyperplasia, reduced cardiomyocyte cell size and, perhaps most prominent, aberrant patterning of the VCS associated with conduction system disease. Overall, our data suggest that EBF1 affects myocardial development by regulating the balance between cellular proliferation and differentiation. Indeed, excessive proliferation is seen in some Ebf1 KO animals well after the immediate postnatal phase, when cell cycle exit should occur. Close coordination of these mutually linked processes is a common theme, and their inverse relationship is a remarkable feature of both development and malignant transformation (Ruijtenberg and van den Heuvel, 2016). Thus, similar to other genetic models of VCS malformation (*Id2*, *Tbx3* and *Nkx2.5*), exuberant cellular proliferation comes at the expense of terminal differentiation (Bakker et al., 2008; Moskowitz et al., 2007; Prall et al., 2007).

Unexpectedly, however, we discovered that EBF1 expression is largely restricted to non-myocytes in the developing and mature heart, including endocardial, perivascular and interstitial cells. Interestingly, non-cell-autonomous regulation of organ growth and differentiation by *Ebf1* may be a mechanism common to other cellular contexts. In kidney development, where generalized knockout of *Ebf1* results in podocyte immaturity and renal dysfunction, podocyte-specific deletion of *Ebf1* does not replicate this phenotype. Instead, conditional knockout in the neighboring mesangial cells (pericyte/smooth muscle) was both necessary and sufficient to affect glomerular maturation (Nelson et al., 2019). Similarly, in bone development, *Ebf1* loss of function results in excessive bone formation, a direct function of osteoblast activity, but osteoblast lineage-specific deletion of *Ebf1* fails to recapitulate the increased bone density seen in the generalized knockout (Zee et al., 2013). Our observation that EBF1 is expressed in perivascular cells and interstitial cells of the heart is also consistent with the previous description of EBF1 expression in pericytes from various tissues (Pagani et al., 2021), and raises the possibility that EBF expression is common to this ubiquitous cell type, with broad effects on multiple tissue types.

In our characterization of the cardiac consequences of *Ebf1* deletion, we focused on the E13.5 developmental timepoint as this is when the myocardial defect is first observed. Additionally, as this timepoint precedes the formation of coronary vasculature and interstitial fibroblasts, it is a relatively simple model in which cardiomyocytes and endocardial cells comprise the vast majority of cells. At this stage, abnormal morphology of the trabecular myocardium is seen, characterized by an excess of cardiac jelly and immature architecture. Cardiac trabeculation is regulated by endocardial-myocardial interactions involving both NOTCH and NRG1 developmental pathways (Del Monte-Nieto et al., 2018). Interestingly, both have also been implicated in VCS development, and suggest a regulatory role for EBF1 in these signaling pathways (Milan et al., 2006; Rentschler et al., 2011, 2012, 2002). The direct transcriptional effects of EBF1 loss of function are seen in the gene profiling of the knockout endocardium, which is most notable for its enrichment of SUZ12 target genes. This finding is corroborated by the ATAC-Seq analysis, which identified changes in chromatin near many of the same target genes. The contribution of chromatin remodeling complexes, such as the PRC and the SWI-SNF complexes, acting in concert with cell type-specific transcription factors to affect the dual regulation of cell cycle and terminal differentiation is well recognized (Ruijtenberg and van den Heuvel, 2016). Although EBF1 is not known to interact with PcG proteins, a role has been described for it in the recruitment of BRG1, a core component of the BAF (SWI/SNF) complex, which antagonizes the repressive effects of PRC2 (Kadoch et al., 2016). Our data support a model in which loss of EBF1 results in an imbalance of these powerful chromatin remodeling complexes, resulting in aberrant PRC2 target gene expression.

Of particular interest among the DEGs are transcription factors that are important for cellular differentiation and proliferation. *Tbx3* is noteworthy due to the known defects in VCS formation observed in null mice, a prominent feature of the *Ebf1* knockout mouse (Bakker et al., 2008; Mohan et al., 2018). Additionally, it is a well described transcriptional target of the PRC2 complex (Oh et al., 2019). Interestingly, the positive effect of TBX3 on cellular differentiation depends on EGR1, which is also a target of PRC2 and EBF1. EGR1 is also an interesting transcriptional target of EBF1 in that it has previously been described as a gene that is highly expressed in adult cardiac fibroblasts relative to fetal or neonatal cardiac fibroblasts, and is a marker of fibroblast maturation (Wang et al., 2020a). The diminished expression of this transcription factor in EBF1-deficient NMCs suggests a defect in fibroblast maturation. Given the defects in cellular differentiation and maturation observed in other EBF1-expressing cell types, we hypothesize that NMC immaturity may be the driving mechanism for the observed myocardial phenotype. Conditional knockout strategies targeting myocardial, endocardial, endothelial and fibroblast lineages cells may clarify the cell type-specific contributions to the observed phenotype.

As a pleiotropic protooncogene, MYC regulates a variety of cellular processes, including cell cycle progression (Amati et al., 1993), growth and differentiation (Melnik et al., 2019), and metabolism (Dang, 2013). Our data suggest that the effect of *Ebf1* loss of function on heart development is mediated through NMC to cardiomyocyte paracrine signaling that non-cell-autonomously induces MYC accumulation in the latter cell type. MYC acts as a central node for proliferation, integrating the signals transduced via RAS-MAPK, Wnt-β-catenin, TGF-β and BMP-SMAD pathways (Dang, 2012), as well as those transduced via exosomes (Borzi et al., 2019). The signaling mechanisms engaged to induce MYC accumulation in EBF1-deficient cardiomyocytes may involve any number of these pathways. The BMP pathway is of particular interest, given our data suggesting an inductive role for BMP signaling in *Ebf1* expression. BMP10 knockout mouse hearts fail to develop normally, with a complete lack of trabecular myocardium, thus establishing its essential role in cardiac growth (Chen et al., 2004). Our data suggest that induction of EBF1 by BMPs may serve as a mechanism to counter the direct effects of BMPs on cardiomyocyte proliferation.

In summary, we have characterized a crucial role for the transcription factor EBF1 in the developing heart and have identified a previously unreported non-cell-autonomous pathway controlling cardiac growth and differentiation. Given the potent ability of EBF1 to regulate cardiomyocyte proliferation, further studies exploring its capacity to influence cell cycling and regeneration in the adult context, especially within diseased myocardium, may be of interest.

## MATERIALS AND METHODS

### Mutant mice

*Ebf1* knockout (Lin and Grosschedl, 1995), *PDGFRα-GFP* (Hamilton et al., 2003) and *Cntn2-EGFP* (Pallante et al., 2010) transgenic mice have been previously described. Mouse lines were maintained in a mixed genetic background. All functional studies were performed on male mice at 3 to 8 weeks of age.

### Histology and immunohistochemistry

Hearts were excised from animals euthanized via either decapitation or cervical dislocation after isofluorane anesthesia. Excised hearts were fixed overnight in 4% paraformaldehyde and cryosections or paraffin wax-embedded sections were prepared and stained as previously described (Pallante et al., 2010). Primary antibodies were directed against EBF1 (R&D Systems; AF5165; 1:50), endomucin (Abcam; AB106100; 1:200), α-actinin (Abcam AB9465; 1:00), troponin T (Fisher Scientific; BD B564766; 1:100), hyaluronic acid binding protein biotinylated (MilliporeSigma; 38591150UG, 1:200), Ki-67 (Abcam; AB16667; 1:100), ERG (Abcam; ab92513; 1:100), c-MYC (Abcam; AB32072; 1:100), GFP (Abcam; AB13970; 1:100), WGA (Thermo Fisher Scientific; W112621; 1:500) and visualized by confocal microscopy (Leica SP5). Image quantification was performed using ImageJ to assess nuclei count per high power field (hpf), total HABP area per hpf, and ratio of Ki-67 (+) nuclei. For Ki-67 staining, transmural images of the left ventricle were used for analysis. EMCN-positive cells were excluded. Two hearts of each genotype were used with multiple transmural stitched images from each heart used for analysis. Cell size quantification was performed using ImageJ to assess for area delineated by WGA. Only cells with cardiomyocyte morphology (rod-shaped with sarcomeres) were included for analysis.

### Echocardiograms and electrocardiograms

Echocardiograms were performed as previously described (Kim et al., 2014) (Vevo; FujiFilm VisualSonics). Surface ECGs were obtained using subcutaneous electrodes attached at the four limbs, as previously described (Gutstein et al., 2003). Mice were anesthetized with inhaled 2% isoflurane. Heart rate was monitored and core body temperature was maintained at 37.5°C using a heat lamp. ECG analysis was performed in an unbiased fashion where 200 beats were analyzed using LabChart 7 Pro version 7.3.1 (ADInstruments). Detection and analysis of P wave, PR interval, QRS wave and QT intervals were set to mouse ECG parameters. Mice with heart rates below 400 bpm were excluded from the analysis.

### VCS whole-mount quantification

Purkinje cell imaging and quantification of EGFP fluorescence were conducted using *Cntn2-EGFP* reporter mice. *Ebf1^−/−^/Cntn2-EGFP* and *Ebf1^+/+^*/*Cntn2-EGFP* mice were generated to study the cardiac conduction system as reported by EGFP. Hearts were excised, immediately placed in ice-cold PBS and fixed in 4% PFA. For imaging of the left VCS, the left ventricular wall was cut open at the center of the free wall. Free wall edges were pinned down using 30-gauge needles to expose the left ventricular septum. For imaging of the right VCS, the anterior region of the right ventricular free wall adjacent to the septum was cut to expose the right ventricular septum and free wall Purkinje fiber network. Bright-field and fluorescent images of the hearts were taken using the Zeiss M2Bio microscope equipped with a Zeiss AxioCam Color camera interfaced with Zeiss AxioVision 2012 software. The area of GFP^+^ Purkinje cells was measured using ImageJ software in regions on the left ventricular septum, right ventricular septum and right ventricular free wall. Quantification was normalized to total area as specified.

### Optical mapping

High-resolution optical mapping experiments were performed as follows: excised hearts from 8- to 12-week-old, male mice were initially perfused with Tyrode's solution to clear blood and stabilize the heart, followed by Tyrode's solution containing 10 μM blebbistatin. Hearts were allowed to recover for 20 min and then stained with the voltage-sensitive dye, Di-4-ANEPPS (Molecular Probes). Light from green LEDs (530 nm; ThorLabs) was used as an excitation source and the emitted light (620 nm long pass) was detected with one high-resolution CMOS camera (Mi-CAM Ultima-L; SciMedia) at 1000 frames per second in bin mode (100×100 pixels) with 14-bit resolution (256×256). Images were processed using a custom software package.

### Flow cytometry

Embryonic day 13.5 wild-type and knockout hearts were dissociated using the Miltenyi MACS Dissociation solutions and trituration (Miltenyi Biotec, 130-098-373). The mitochondrial dye tetramethylrhodamine methyl ester perchlorate (TMRM 50 nm; Invitrogen) was used to identify cardiomyocytes. Indirect labeling using PECAM1 antibody (RandD Systems; AF3628) was performed to identify endocardial cells. FACS (Beckman Coulter MoFlo) was performed as previously described (Kim et al., 2014). For FACS analysis of VCS cell proportion, VCS cells (TMRM^+^/CNTN2-EGFP^+^) were counted and expressed as a percentage of total cardiomyocytes (TMRM^+^/CNTN2-EGFP^−^).

### Gene transcriptional profiling

RNAs were extracted from isolated cells using the RNeasy Mini kit (Qiagen). Sequencing libraries were prepared using the TruSeq RNA Library Prep Kit v2 (Illumina). Samples were sequenced 50 bp paired-ended at 10 million to 20 million reads per replicate on an Illumina HiSeq. 2500 instrument. Library preparation and sequencing were performed at New York University School of Medicine Genome Technology Center. All the reads were mapped to the mouse reference genome (mm10) using the STAR aligner (v2.5.0c) (Dobin et al., 2013). Alignments were guided by a Gene Transfer File (GTF, version GRCm38.74) and the mean read insert sizes and their standard deviations were calculated using Picard tools (v.1.126) (http://broadinstitute.github.io/picard/). The read count tables were generated using HTSeq (v0.6.0) (Anders et al., 2015), normalized based on their library size factors using DESeq. 2 (v3.0) (Love et al., 2014) and differential expression analysis was performed. The Read Per Million (RPM) normalized BigWig files were generated using BEDTools (v2.17.0) (Quinlan and Hall, 2010) and bedGraphToBigWig tool (v4), and downstream statistical analyses and generating plots were performed in R environment (v3.1.1) (http://www.r-project.org/). ChEA3 analysis was performed as previously described (https://maayanlab.cloud/chea3/). Gene sets obtained from RNA-Seq and ATAC-Seq analyses were input and transcription factors with significantly enriched binding sites were rank ordered according to *P*-value. Data are represented as odds ratios, −log10 *P*-value, and combined score.

### Gene set enrichment analysis (GSEA)

Ranked list file format (rnk) files were made using log2 fold changes of all the genes and GSEA was performed using GSEA tool (https://www.gsea-msigdb.org/gsea/index.jsp) for the following gene sets (gmt file): c2.cp.kegg.v7.0.symbols.gmt (‘KEGG pathways’), c5.mf.v7.0.symbols.gmt (‘molecular function’), c5.bp.v7.0.symbols.gmt (‘biological process’), h.all.v7.0.symbols.gmt (‘immunological signature’) and c7.all.v7.0.symbols.gmt (‘cancer hallmark’).

### qPCR

RNA was reverse-transcribed to cDNA using the Maxima First Strand cDNA Synthesis Kit from Thermo Scientific. qPCR was performed with a SYBR qPCR Kit (Qiagen) on a StepOne Real-Time PCR System (Applied Biosystems). qPCR probes were purchased from Origene. Differences between samples and controls were calculated based on the ΔΔCT method (Livak and Schmittgen, 2001) and normalized to GAPDH. Statistical significance was tested using one-way ANOVA (and nonparametric), *P*<0.05 were considered statistically significant.

### ATAC-seq

Embryonic day 13.5 NMCs were isolated via enzymatic digestion using the Miltenyi Biotec heart dissociation kit (Miltenyi Biotec, 130-098-373). Cells were preplated for 2 h to isolate NMCs. NMCs were cultured in serum-free DMEM with supplements. 50K cells were subjected to the transposition reaction, as previously described (Buenrostro et al., 2015), using Tagment DNA Enzyme 1 (TDE1) (Illumina, 15027865) along with Tagment DNA Buffer (Illumina, 15027866). The amplified library was purified using the MinElute Reaction Cleanup Kit (Qiagen, 28204).

### Western blots

E13.5 mouse hearts were excised, and ventricles isolated and rinsed in PBS then lysed in RIPA buffer with protease and phosphatase inhibitors. Clarified lysates were run on 4–20% precast polyacrylamide gradient gels (Invitrogen) and transferred to nitrocellulose (Bio-Rad) overnight at 4°C. Nitrocellulose membranes were incubated in blocking buffer consisting of TBS with Tween-20 (0.05%) and 5% nonfat dry milk. Membranes were then incubated with primary antibodies directed against c-Myc (Invitrogen; MA1-980; 1:500) and vinculin (Abcam; 130007; 1:1000) diluted in 5% nonfat dry milk in TBS-T (TBS with 0.05% Tween 20) overnight at 4°C, followed by wash steps and incubation with donkey anti-mouse secondary antibodies (LI-COR Biosciences; 926-68072; 1:5000). Antigen complexes were visualized and quantified with the Odyssey Imaging 1227 System (LI-COR Biosciences).

### Embryonic heart culture assays and inhibitor assay

E9.5 C57b/6 hearts were harvested and cultured in DMEM containing 1% FBS, penicillin and streptomycin (GIBCO/Invitrogen) in 24-well culture plates. Vehicle control, BMP7 (10 ng/ml) (Bramlage et al., 2010), BMP10 (5 ng/ml) (Mitrofan et al., 2017), FGF1 (50 ng/ml), epiregulin (20 ng/ml) (Kong et al., 2014), TGFβ (10 ng/ml) (Bramlage et al., 2010), angiopoietin 1 (250 ng/ml) (Patschan et al., 2013), neuregulin 1 (2.5 nM) (Rentschler et al., 2002), IGF1 (100 ng/ml) (Sprynski et al., 2009), LDN (1 µM) (Galvin-Burgess et al., 2013) or gremlin 2 (100 ng/ml) (Nilsson et al., 2014) was added to each well. Medium in both conditions was replaced every 12 h. Cultures were maintained for up to 48 h. Non-beating cultures were excluded from analysis.

### Study approval

All protocols conformed to the Association for the Assessment and Accreditation of Laboratory Animal Care and the NYU School of Medicine Animal Care and Use Committee.
